# Ag-doped ZnO nanorods embedded reduced graphene oxide nanocomposite for photo-electrochemical applications

**DOI:** 10.1098/rsos.181764

**Published:** 2019-02-06

**Authors:** Farheen Khurshid, M. Jeyavelan, M. Sterlin Leo Hudson, Samuthira Nagarajan

**Affiliations:** 1 Department of Chemistry, Central University of Tamil Nadu, Thiruvarur, India; 2 Department of Physics, Central University of Tamil Nadu, Thiruvarur, India

**Keywords:** photocurrent, ZnO, reduced graphene oxide, photocatalyst, methyl orange

## Abstract

In this paper, the Ag-doped zinc oxide nanorods embedded reduced graphene oxide (ZnO:Ag/rGO) nanocomposite was synthesized for photocatalytic degradation of methyl orange (MO) in the water. The microstructural results confirmed the successful decoration of Ag-doped ZnO nanorods on rGO matrix. The photocatalytic properties, including photocatalytic degradation, charge transfer kinetics and photocurrent generation, are systematically investigated using electrochemical impedance spectroscopy (EIS), photocurrent transient response (PCTR) and open circuit voltage decay (OCVD). The results of photocatalytic dye degradation measurements indicated that ZnO:Ag/rGO nanocomposite is more effective than pristine ZnO to degrade the MO dye, and the degradation rate reached 40.6% in 30 min. The decomposition of MO with ZnO:Ag/rGO nanostructure followed first-order reaction kinetics with a reaction rate constant (*K*_a_) of 0.01746 min^−1^. The EIS, PCTR and OCVD measurements revealed that the Ag doping and incorporation of rGO could suppress the recombination probability in ZnO by the separation of photo-generated electron–hole pairs, which leads to the enhanced photocurrent generation and photocatalytic activity. The photocurrent density of ZnO:Ag/rGO, ZnO/rGO and pristine ZnO are 206, 121.4 and 88.8 nA cm^−2^, respectively.

## 1. Introduction

In day-to-day life, water contamination is one of the serious issues for humankind and the environment. Water contamination occurs due to many factors such as wastages from industries, including organic dyes, fertilizers, hydrocarbons, pesticides (phenols and biphenyls), plasticizer, detergents, oils, greases, pharmaceuticals, proteins, carbohydrates, organic pollutants [1]. The photocatalytic degradation process has been extensively used for the degradation of organic dyes in the water. Owing to the excellent physical and chemical properties, such as low toxicity, electrochemical stability, super oxidative capacity and wide band gap, metal oxides are the most suitable candidates for the photodegradation [2]. Among them, ZnO is a well-known abundant inorganic wide bandgap (3.37 eV) semiconductor and it is considered as one of the most superior metal oxides, which is playing a vital role in many applications such as photocatalytic activity, solar cells, gas sensors, electrochemical capacitors and batteries [3–6]. Owing to the excellent physical and chemical properties, such as high electrochemical stability, super oxidative capacity and low toxicity, ZnO is a promising material for the application of photocatalytic activity. Therefore, among the other metal oxides, ZnO is the first and widely used material in heterogeneous photocatalysis [7–10]. Although it has numerous advantages in the photocatalytic process, the fast recombination of photo-excited charge carriers in ZnO hinders the photocurrent generation and photocatalytic efficiency. To improve the electrical and optical properties for enhancing the photocatalytic efficiency of ZnO, elite studies are going on the modification ZnO with metal [11] and non-metal [12] additions. In a non-metal addition, graphene is a promising choice for the researchers, because of its unique properties like high mobility (10 000 cm^2^ V^−1^), [13] high electrical conductivity (3.49 × 10^2^ S cm^−1^), [14] large surface area (2000 m^2^ g^−1^) [15] and high thermal conductivity (3000–5000 W m^−1^ K^−1^) [16]. Owing to these fascinating properties, graphene can be used as an electron transporting medium in photocurrent and photocatalytic process [17]. As a result, the incorporation of graphene with ZnO leads to improve the charge separation efficiency of metal oxide for the benefit of enhanced photocurrent and photocatalytic activity. Herring *et al.* have prepared the ZnO/rGO (GZ) nanocomposite for the application of photocatalytic dye degradation [18]. The enhanced photocatalytic activity of ZnO/rGO is ascribed to the photo-generated electron transfer from the conduction band ZnO to graphene. Ahmad *et al.* investigated the photocatalytic activity of ZnO/graphene nanocomposite and they observed approx. 100% photocatalytic dye degradation efficiency under the visible light irradiation for 90 min [19]. Subsequently, metal ion doping and metal nanoparticle decoration are alternative choices for changing the electrical and optical properties of ZnO [20]. Many researchers have extensively studied the photocatalytic activity of metal-modified ZnO and nanoparticle-decorated ZnO [21]. However, the decoration of ZnO with metals has an excellent influence on the photocatalytic activity [22]. Basically, the incorporation of metal in the crystal lattice leads to the native defects and oxygen vacancies in ZnO due to the mismatch in the ionic radius carrier concentration of dopant. These defects and oxygen vacancies act as the sub-band gap donor sites, which also generate the photo electrons during the light illumination. Besides absorbing the light, the defects and vacancies also act as the recombination centre for photo-induced electrons, which improves the diffusion of photo-generated electrons in ZnO, thus facilitating the charge carrier separation and thereby reducing the recombination rate [23]. In this context, Wang *et al.* have reported that the photocatalytic activity of ZnO was enhanced due to silver doping. Reports say that the silver can effectively trap the photo-generated electrons from ZnO [24]. Furthermore, Ghosh *et al.* opened the path of doped ZnO with copper and made as a copper-doped ZnO nanowire for high photocatalytic activity and they have observed quick relaxation time due to the fast trapping of photo-generated electrons by the dopants [25]. Debasmita Sardar *et al.* synthesized ZnO/Ag with different ratios of Ag and they observed a highest photo degradation of 56% for ZnO/Ag [26]. In this study, the Ag-doped zinc oxide nanorods embedded reduced graphene oxide (ZnO:Ag/rGO) nanocomposite was synthesized by hydrothermal method. The microstructural studies were carried out to analyse the properties of Ag-doped ZnO nanorods on rGO matrix. The electrochemical properties, including the photocatalytic activity, charge transfer kinetics, photocurrent generation, are systematically investigated from photocatalytic degradation of methyl orange (MO) dye, electrochemical impedance spectroscopy (EIS), photocurrent transient response (PCTR) and open circuit voltage decay (OCVD).

## 2. Material and methods

### 2.1. Chemicals

Graphite powder (200 mesh) was purchased from Alfa Aesar. ZnO, zinc acetate, silver nitrate and zinc nitrate were procured from Sigma Aldrich.

### 2.2. Synthesis of ZnO:Ag nanorods

The Ag-doped zinc oxide nanorod was prepared by hydrothermal method. To synthesize the Ag-doped ZnO nanorods, the aqueous solution of 0.5 M zinc nitrate (Zn(NO_3_)_2_·6H_2_O) and 0.05 M silver nitrate (AgNO_3_) were mixed under constant stirring for 1 h at ambient temperature. To maintain the pH value of 9.5, few drops of ammonium hydroxide were slowly added into the solution. The final solution was then transferred into Teflon-lined autoclave followed by vigorous stirring (for 30 min). Thereafter, the autoclave was sealed and heated up to 200°C for 6 h. The final product was washed several times with ethanol and DI water and dried at 60°C in a hot air oven overnight. The EDAX and SEM analyses were conducted to study the elemental composition and morphology of synthesized samples.

### 2.3. Synthesis of ZnO:Ag/rGO nanocomposite

The GO was synthesized by modified Hummer's method which is given in the electronic supplementary material. The nanocomposite was prepared in such a way that a 100 mg of GO was initially dispersed in 50 ml DI water under ultra-sonication for 20 min. The solution was constantly stirred for 1 h and ZnO:Ag solution (20 mg/40 ml) was added dropwise. Thereafter, the final solution was vigorously stirred and subsequently transferred to the Teflon-lined autoclave which was sealed and heated up to 400°C for 4 h. The final product was collected after the centrifugation at 4000 r.p.m. for 10 min, the obtained product was repeatedly washed with ethanol and DI water and dried at 45°C for 12 h. The structural and micro-structural, electrochemical properties of the as-prepared nanocomposite were investigated.

### 2.4. Characterization

The structural analysis of the samples was carried out by using powder X-ray diffractometer (PAN analytical X'Pert PRO) equipped with X'Celerator position sensitive detector using Cu K*α* radiation of wavelength *λ* = 1.5401 Å. Microstructural analysis of the samples was characterized by using JEOL (SEM and TEM). The UV–vis absorption spectra were obtained between 200 and 800 nm using Shimadzu UV-1800 spectrophotometer, equipped with quartz cuvettes with optical path lengths of 1 cm. The Raman spectra of the samples were recorded by using EZ Raman-N-785 spectrometer. The DC *I*–V characteristics of samples were analysed between −5 and +5 V using a Keithley 2636B source meter.

### 2.5. Photo-electrochemical measurements

To understand the charge transportation kinetics, photocurrent generation and recombination kinetics of the samples, the electrochemical studies such as EIS, PCTR and OCVD measurements were carried out by using Biologic VSP-300 electrochemical workstation with the standard three-electrode configuration (working, reference and counter electrode). The photo anode was used as a working electrode and it was prepared by coating the nanocomposite film (an active area of 1 cm^2^) on the FTO substrate (sheet resistance = 7 Ω sq^−1^) using doctor blade technique and dried at 300°C for 30 min. The standard calomel electrode and platinum wire are used as the reference and counter electrode, respectively. 0.1 M of Na_2_SO_4_ was used as an electrolytic medium for the electrochemical analysis. The electrochemical impedance spectral (EIS) properties of the samples were analysed in the frequency range from 500 mHz to 1 MHz under dark and UV light irradiation (300 W mercury lamp). The PCTR measurements were done under UV irradiation (300 W mercury lamp) with 5 min ON/OFF cycle at an input voltage of 0.5 V. The OCVD characteristics were measured as a function of time in which the voltage growth was monitored under UV irradiation for 60 s and then the voltage decay was recorded in the dark for 90 s. The photocatalytic degradation activity of ZnO:Ag/rGO, ZnO/rGO and pristine ZnO was monitored by measuring the percentage degradation of MO dye. The photocatalytic solutions were prepared by using a photo-reactor, in which a 300 W mercury lamp was used as a UV source. A 50 mg of photocatalyst was dispersed in 100 ml MO solution (10 mg l^−1^). Prior to the UV irradiation, the suspensions were sonicated for 15 min in the dark to maintain the adsorption–desorption equilibrium. To achieve the homogeneity during the photocatalytic measurement, the photo-reactor was placed on a magnetic stirrer in the dark. A 3 ml of suspension was taken out from the photo-reactor at regular time intervals and then the absorption of MO was monitored under visible light (at *λ* = 480 nm) by using Shimadzu UV-1800 spectrophotometer. The percentage of photocatalytic degradation was calculated from the following equation [27]:

$$\text{\%}\mathrm{Degradation}=\frac{A_{0}-A}{A_{0}}\;\;\times 100\text{\%}.$$

where *A*_0_ is the absorbance of MO before UV irradiation.

## 3. Result and discussion

**Figure 1.**
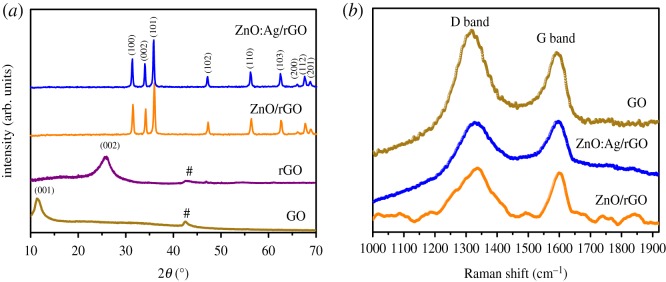
(*a*) XRD spectra of GO, rGO, ZnO/rGO and ZnO:Ag/rGO, (*b*) Raman spectra of GO, ZnO/rGO and ZnO:Ag/rGO.

The X-ray diffraction spectra of ZnO:Ag/rGO, ZnO/rGO, rGO and GO are presented in figure 1*a*. The characteristic diffraction peak of graphene oxide (GO) is observed at 11.3° corresponding to the *d*-spacing of 0.76 nm, which is 2.3 times larger than the *d*-spacing of graphite (0.33 nm). The enlarged *d*-spacing of GO originated from the intercalation of oxygen-containing functional groups (discussed and given in the electronic supplementary material, §4) between the graphitic interlayers distance due to the strong chemical oxidation treatment on graphite. After the oxidation, a very small fraction of unreacted graphitic phases also presents in GO, it can be clearly observed at 42.5°. The thermal reduction at 400°C on GO causes large inter-planar spacing due to the elimination of oxygen-containing functional groups from the carbon interlayers. Therefore, the diffraction peak of GO shifted from 11.3° to 25.4°. Hence from the diffraction spectra of GO and rGO, the peak shifting towards lower angle reveals the successful modification of sp^2^ (graphite) into sp^3^ (GO) hybridized carbon structure. But, the peak shifting towards the higher angle confirms the transformation of GO into rGO. The diffraction peaks of ZnO are observed in ZnO/rGO and ZnO:Ag/rGO as shown in figure 1*a*. All these peaks are indexed to be the hexagonal Wurtzite structure of ZnO and agreed well with standard data sheet (JCPDS-36-1451) [28]. Figure 1*b* shows the Raman spectra of ZnO:Ag/rGO, ZnO/rGO and GO. There are two prominent peaks (G-band and D-band) for all samples. The G-band observed at 1592 cm^−1^ is attributed to the presence of sp^2^ hybridized carbon structure, whereas, the D-band at 1318 cm^−1^ occurs from the localized vibrational modes of oxygen functionalities [29,30]. Raman spectroscopy is a useful method to characterize the degree of structural disorder in the samples by correlating the intensity ratio (ID/IG). Therefore, the degree of disorder is proportional to the *I*_D_/*I*_G_ value. The estimated *I*_D_/*I*_G_ values from the Raman spectra are 1.10, 0.99 and 1.01 respectively, it reveals that the structural disorder of ZnO:Ag/rGO and ZnO/rGO is less than that of GO. The reason behind this is that during the thermal reduction on ZnO:Ag/rGO and ZnO/rGO, the regeneration occurs in the carbon structure from sp^3^ into sp^2^, which leads to regaining the order in the carbon structure, as a result the intensity of the D-band is less than that of G-band.

**Figure 2.**
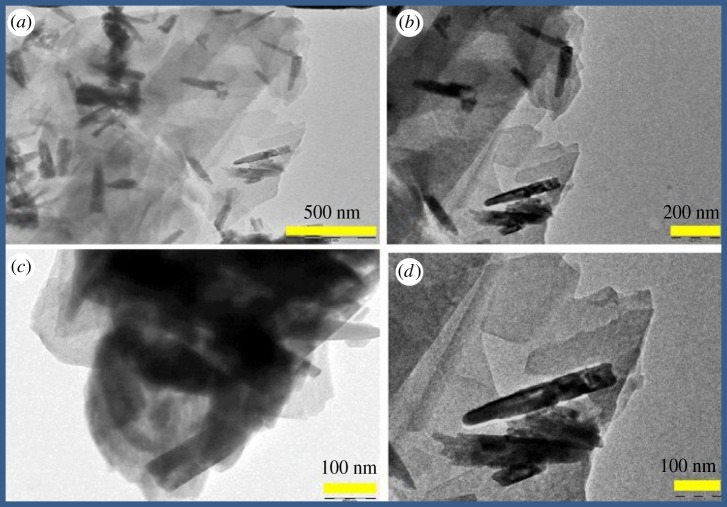
(*a*–*d*) TEM images of ZnO:Ag/rGO.

**Figure 3.**
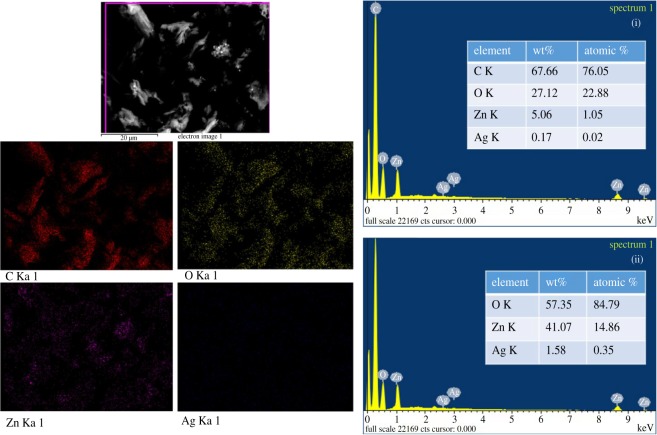
SEM morphology, elemental mapping and EDAX analysis of ZnO:Ag/rGO. The atomic and weight percentages of the elements in ZnO:Ag/rGO were calculated with C and O (i) and without C and O (ii).

**Figure 4.**
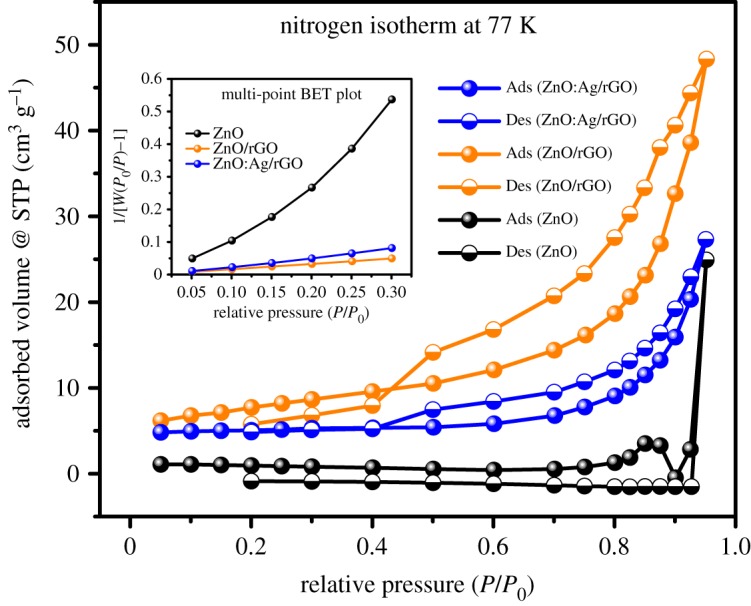
BET curves and the insert graph represents the multi-point plots of ZnO:Ag/rGO, ZnO/rGO and ZnO.

The typical TEM images of ZnO:Ag/rGO are shown in figure 2*a–d*. It is identified from the image in low magnification (figure 2*a*,*b*) that the zinc oxide nanorods are randomly distributed on rGO sheets. The ZnO nanorods are observed very clearly from the high magnification images (figure 2*c*,*d*). It has been estimated that the diameter and the length of the nanorods are about 50 ± 5 and 300 ± 10 nm, respectively. TEM images reveal that the rGO sheets are with the ZnO nanorods. Furthermore, the rGO sheets overlap with each other and form a three-dimensional network structure on ZnO nanorods, which provides a fast conducting path for the photo-induced electrons of ZnO nanorods. It leads to the better charge transportation in the photocatalytic process. The random distribution of ZnO nanoparticles on rGO is shown in electronic supplementary material, figure S2. The surface morphologies of ZnO:Ag/rGO are shown in figure 3. The SEM morphology shows that the ZnO:Ag/rGO forms a flake-like structure. The EDAX analysis confirms the presence of C, O, Zn and Ag in the composite and also the weight percentage of Ag is 0.17%. But, the existence of Ag is not found in ZnO/rGO as shown in electronic supplementary material, figure S2. The elemental mapping of ZnO:Ag/rGO, as shown in figure 3, confirms the coexistence of all elements of the composite. BET surface area of the samples was reordered under N_2_ adsorption/desorption isotherm at 77 K. The corresponding isotherms and multi-point fit plots of ZnO, ZnO/rGO and ZnO:Ag/rGO are shown in figure 4. The multi-point plot reveals that the ZnO, ZnO/rGO and ZnO:Ag/rGO samples have a surface area of 2.3605, 26.625 and 15.750 m^2^ g^−1^, respectively. As observed from the BET measurement, the ZnO has very low surface area. However, the surface area increases after the incorporation of rGO in ZnO and ZnO:Ag. The isotherm of all the samples resembles the type IV (IUPAC) isotherm and it indicates that the materials are mesoporous. The cumulative pore volumes of ZnO, ZnO/rGO and ZnO:Ag/rGO are 0.0385, 0.0671 and 0.0422 cm^3^ g^−1^, respectively. The calculated BET parameters of the samples are given in table 1.

**Table 1.** BET parameters of ZnO:Ag/rGO, ZnO/rGO and ZnO.

samples	surface area (m^2^ g^−1^)	pore volume (cm^3^ g^−1^)	pore size (nm)
ZnO	2.3605	0.0385	28.27
ZnO/rGO	26.625	0.0671	8.426
ZnO:Ag/rGO	15.750	0.0422	10.726

**Figure 5.**
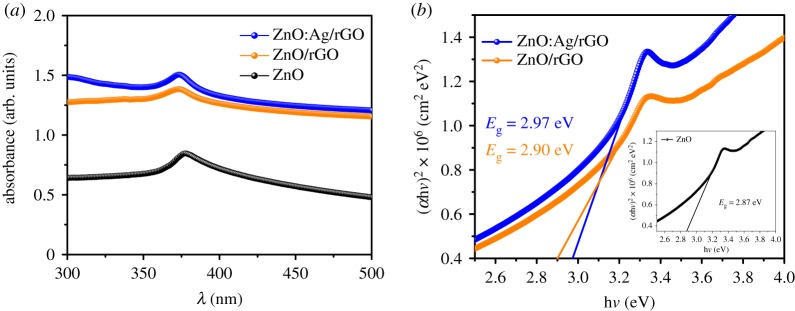
(*a*) UV spectra of ZnO:Ag/rGO, ZnO/rGO and ZnO, (*b*) Tauc plot of ZnO:Ag/rGO, ZnO/rGO and ZnO.

**Figure 6.**
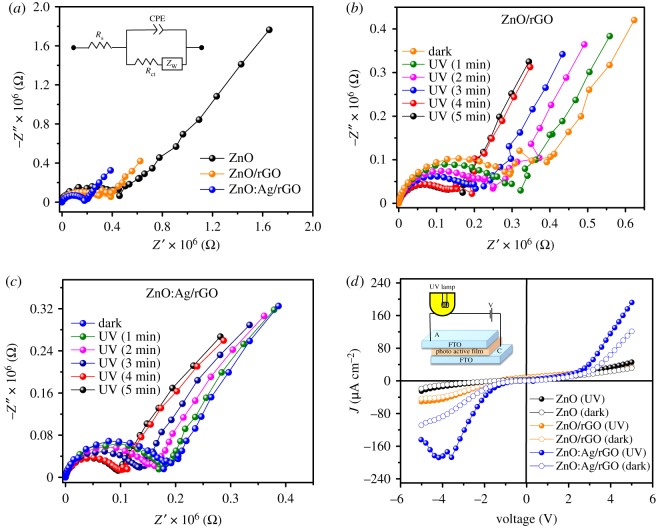
(*a*) Impedance spectra of ZnO:Ag/rGO, ZnO/rGO and ZnO, (*b*) impedance spectra of ZnO/rGO, (*c*) impedance spectra of ZnO:Ag/rGO, (*d*) *I*–*V* characteristic of ZnO:Ag/rGO, ZnO/rGO and ZnO.

The UV–vis absorption spectra of ZnO:Ag/rGO, ZnO/rGO and pristine ZnO are presented in figure 5*a*. The optical absorption maxima of all samples are observed between 372 and 377 nm, absorption maxima of ZnO:Ag/rGO (372 nm) and ZnO/rGO (375 nm) are blue-shifted from the pristine ZnO (377 nm). The optical bandgap of the samples is calculated by using *αhν* = *A*(*hν* − *E*_g_)^1/2^, where *α* is an absorption coefficient, *hν* is the energy of the photon, *A* is a constant, *E*_g_ is the bandgap energy of the sample and 1/2 defines the direct allowed transition. The optical bandgap of the samples is calculated by the extrapolation of a linear portion in the plot of (*αhν*)^2^ against *hν* presented in Figure 5*b*. The calculated band gaps of ZnO:Ag/rGO, ZnO/rGO and pristine ZnO are 2.97, 2.90 and 2.87 eV, respectively, indicating that the bandgap of ZnO is found to increase after the addition of rGO. This is because the thermal reduction on ZnO/GO composite can create oxygen vacancies in ZnO [31]. After Ag doping, a blue shift is observed in the bandgap energy of ZnO/rGO, attributed to the phenomenon called Moss–Burstein effect caused by the generation of electrons due to oxygen vacancies [32]. The substitution of Ag^2+^ in Zn^2+^ lattice, thus creating more oxygen vacancies and high electron density due to electronegativity and variation in the ionic radius between Ag^2+^ and Zn^2+^. This high electron density leads to the lifting of Fermi level into the conduction band of the semiconductor results in widening of the bandgap in ZnO. The similar effect has been also reported for Al-doped ZnO, Sn-doped In_2_O_3_, Cd-doped In_2_O_4_ and Mg-doped ZnO [32–35]. The electrochemical impedance spectroscopy (EIS) measurements were carried out to understand the electrochemical charge transportation properties of ZnO:Ag/rGO, ZnO/rGO and pristine ZnO. The impedance spectra of the samples were recorded under both dark and UV irradiation, the respective Nyquist plots and its equivalent circuit are shown in figure 6*a*. The equivalent circuit consists of a series resistance (*R*_s_), a charge transfers resistance (*R*_ct_), Warburg impedance (*Z*_w_) and a capacitor. The electrochemical charge transfer resistances of ZnO:Ag/rGO, ZnO/rGO and pristine ZnO calculated from the semicircle of the impedance curve in the high-frequency region are 0.17, 0.38 and 0.46 MΩ, respectively. As observed from figure 6*a*, the radius of the semicircle of the impedance curve decreases in the order of ZnO > ZnO/rGO > ZnO:Ag/rGO. The smaller the *R*_ct_ of the ZnO:Ag/rGO leads to the better charge transportation at the interface between the working electrode (photo anode) and electrolyte. As the presence of rGO and Ag in ZnO:Ag/rGO nanocomposite promotes the mobility of charge carriers in the working electrode, the Ohmic resistance at the interface between the working electrode and electrolyte is reduced. In addition, the series resistance (*R*_s_) determined from the Nyquist curve illustrates that the intrinsic conductivity of ZnO is enhanced by the incorporation of rGO and Ag. Figure 6*b*,*c* represents the photo-dependent electrochemical impedance spectra of ZnO/rGO and ZnO:Ag/rGO. The photo-dependent impedance spectra were recorded under the illumination of UV light for 1 to 5 min. It is observed from figure 6*b* that the radius of the semicircle of the impedance curve is reduced when the working electrode was exposed by the UV irradiation and the corresponding *R*_ct_ is decreasing from 0.3 (dark) to 0.16 MΩ (UV), indicating that the presence of rGO is enhancing the mobility of photo-generated charge carrier in the photo anode (ZnO/rGO). The opto-electrochemical property of ZnO/rGO is further improved by the Ag doping. The representative impedance curves of ZnO:Ag/rGO (figure 6*c*) show the enhanced optical response under the UV irradiation, in which the *R*_ct_ value decreases from 0.18 (dark) to 0.09 MΩ (UV). It emphasize that the ZnO:Ag/rGO can be used as a promising material for the opto-electrochemical applications. The DC I–V characteristics of ZnO:Ag/rGO, ZnO/rGO and pristine ZnO films were analysed in the dark and UV with an interval of 5 min. The measurements were recorded between the sweep voltage of −5 and +5 V. Figure 6*d* represents that the photocurrent as well as the dark current properties of ZnO is improved due to the addition of rGO and Ag doping with ZnO. The current density of the ZnO:Ag/rGO is found to increase from 120 to 192 µA cm^−2^ after the illumination of UV for 5 min. Besides light absorption, the photo-induced charge carrier transportation and separation are also considered to be the key factors, which will enhance the photocurrent generation and photocatalytic activity of ZnO. Many researchers also reported that the photocurrent and photocatalytic performance of ZnO can be improved by the incorporation of rGO [36–42]. Graphene is an excellent acceptor candidate due to its unique two-dimensional π-conjugation structure [36], it results in the fast transportation of photo-induced charge carriers from semiconductor into graphene via percolation mechanism [43]. But, photo-electrochemical properties can also be enriched by the addition of impurities (such as Ag, Fe, Ni and Pd) into the intrinsic ZnO via the doping process [27,44–46]. The charge carrier concentration of doped atom (impurity) and the interstitial defects which are caused by the doped atom will improve the photocurrent and photocatalytic efficiency of a semiconductor material. This study reveals that the photocurrent properties of ZnO can be markedly improved by the addition of rGO and Ag doping.

**Figure 7.**
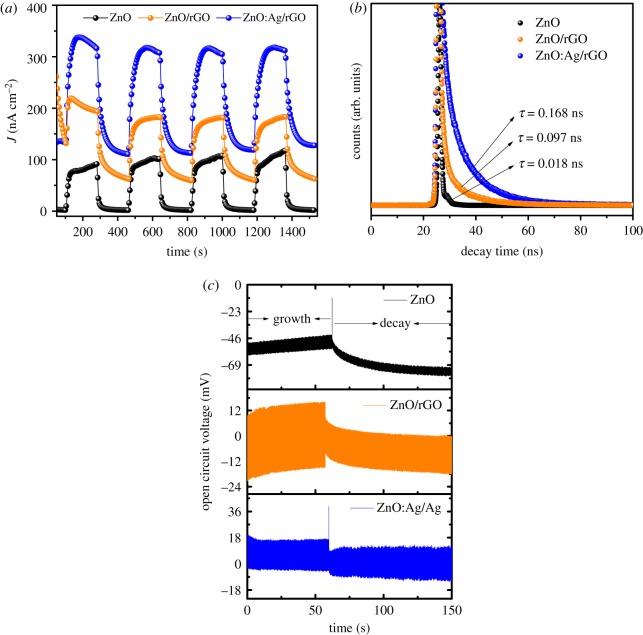
(*a*) PCTR, (*b*) TCSPC and (*c*) OCVD curves of ZnO:Ag/rGO, ZnO/rGO and ZnO.

To explore the photocurrent properties of ZnO:Ag/rGO photocatalyst, the PCTR measurement was carried out by applying 0.5 V between working and reference electrodes and the current has been measured between working and counter electrodes. For comparison, the similar measurement was also carried out on the ZnO/rGO and pristine ZnO. The photocurrent density of samples was measured as a function of time under the illumination of UV light with ON/OFF cycles at an interval of 5 min. The PCTR curves of ZnO:Ag/rGO, ZnO/rGO and pristine ZnO are shown in figure 7*a*. As observed from figure 7*a*, the photocurrent response rapidly increased and reached the saturation point once the light is switched ON. It can be clearly seen that the photocurrent response of ZnO/rGO is increased due to the addition of rGO; this result agreed well with previous works [39]. Significantly, the ZnO:Ag/rGO shows superior photocurrent generation, compared to ZnO/rGO and pristine ZnO, indicating that the photo-conversion efficiency of ZnO/rGO is further improved after Ag doping. The excess carrier concentration, owing to the ionization of donor (Ag), leads to an increase in the n-type conductivity as well as photocurrent density of ZnO/rGO. In addition to that, the lattice defects caused by the dopant ions are also contributing to the photocurrent generation [27,46]. The photocurrent density of ZnO:Ag/rGO is 206 nA cm^−2^, which is approximately 2.3 times higher than that of pristine ZnO. This enhancement in the photocurrent density of ZnO:Ag/rGO reveals that the photo-generated charge carrier separation efficiency of the sample is promoted after Ag doping along with the addition of rGO, resulting in a decrease in its recombination probability. The evidence for the low recombination property of ZnO:Ag/rGO is confirmed from the OCVD measurement (figure 7*c*). In a semiconductor, the photo-induced electrons in the conduction band and holes in the valence band are developing a potential (open circuit voltage). This potential growth occurs due to the excitation of charge carriers, whereas the potential drop occurs due to the recombination of photo-induced charge carriers. Therefore, the magnitude of the potential drop is proportional to the lifetime of the photo-generated charge carriers in the semiconductor. Compared to pristine ZnO and ZnO/rGO, the ZnO:Ag/rGO is displaying a lower potential drop (Δ*V* = *V*_growth_ − *V*_decay_) (−3.63 mV). It represents that the photo-induced charge carriers in ZnO are quickly trapped by rGO, which leads to the minimization of potential drop via reducing the carrier recombination rate and thereby increasing the lifetime of photo-induced charge carriers [47,48]. The improved lifetime of photo-generated charge carrier in ZnO:Ag/rGO is calculated from the time-correlated single photon count (TCSPC) measurement. As observed from figure 7*b*, the excited carrier lifetime of ZnO/rGO is found to increase up to 0.168 ns after Ag doping. On the pristine ZnO, the photo-generated charge carriers would undergo fast recombination, thus hindering the electron transfer from anode to cathode, which results in less photocurrent generation. The high potential drop (Δ*V* = −25.1 mV) in the open circuit voltage of pristine ZnO also confirms its fast recombination behaviour. As presented in figure 7*a*, the dark current density (current density in the absence of light) of ZnO:Ag/rGO is found to be 133 nA cm^−2^, which is approximately 65 times higher than that of pristine ZnO and approximately two times than that of ZnO/rGO, thus indicating that the donor impurity (Ag) and the presence of rGO has effectively changed the carrier concentration in ZnO and thereby increasing its electrical conductivity. From PCTR and OCVD measurements, the calculated values of ZnO:Ag/rGO, ZnO/rGO and pristine ZnO are shown in table 2. The average lifetime (〈*τ*〉 = *Σ*_i_ *A*_i_*τ*_i_) of photo-induced charge carrier in ZnO:Ag/rGO, ZnO/rGO and pristine ZnO is calculated by using third-order exponential decay fit and the corresponding values are also shown in table 2.

**Table 2.** PCTR, OCVD and TCSPC spectral analysis of ZnO:Ag/rGO, ZnO/rGO and ZnO.

sample	PCTR				OCVD			TCSPC
	ON/OFF duration min	*J*_min_ nA cm^−2^	*J*_max_ nA cm^−2^	photocurrent density (Δ*J* = *J*_max_ − *J*_min_) nA cm^−2^	*V*_growth_ mV	*V*_decay_ mV	Δ*V* mV	lifetime (*τ*) ns
ZnO:Ag/rGO	5	132	338	206	−0.15	−3.78	−3.63	0.168
ZnO/rGO	5	60.7	182.1	121.4	−0.48	−8.83	−8.35	0.097
ZnO	5	1.6	90.4	88.8	−49.1	−74.2	−25.1	0.018

**Figure 8.**
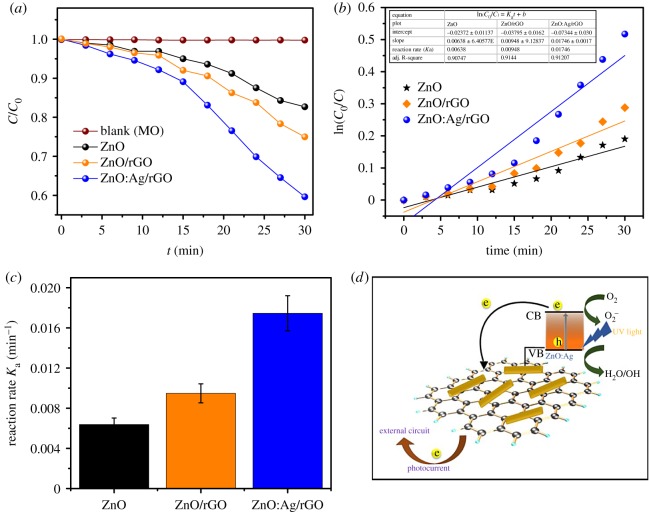
(*a*–*c*). Photocatalytic activity of ZnO:Ag/rGO, ZnO/rGO and ZnO, (*d*) schematic diagram of the photocatalytic mechanism of ZnO:Ag/rGO.

The photocatalytic activity of ZnO:Ag/rGO, ZnO/rGO and pristine ZnO was evaluated by the degradation of MO under UV irradiation. The UV absorbance spectra of the photocatalysts (ZnO, ZnO/rGO and ZnO:Ag/rGO) are given in the electronic supplementary material, figure S4. The normalized changes in the concentration (C/C_0_) of MO during photodegradation are proportional to the normalized maximum absorbance (A/A_0_). As represented from figure 8*a*, after UV irradiation for 30 min, the degradation of MO without photocatalysts (blank) remains unchanged. The ZnO:Ag/rGO shows a remarkable enhancement in photodegradation of MO compared with ZnO/rGO and pristine ZnO, which took only 30 min to degrade 40.6% of MO. But, the ZnO/rGO and pristine ZnO could degrade only 25.2% and 17.3% of MO in the same interval. This faster degradation rate of MO under UV irradiation using ZnO:Ag/rGO is attributed to the increase in the charge density and then the defect sites which are caused by Ag doping, leading to an enhanced optical absorption and carrier generation. In addition to that, the presence of rGO in the nanocomposite makes a three-dimensional conducting network on the Ag-doped ZnO nanorods, which plays a vital role in the transportation of photo-induced charge carrier.

Therefore, the addition of rGO and Ag induced shallow donor and acceptor states which act as an electron or hole trap and thereby reduce the carrier recombination in ZnO. Hence, the photo-generated electrons are involved in a reaction with O_2_ and form radicals such as •O_2_^−^, •OH for the degradation of MO. But holes in the valence band of ZnO can take part in the redox reactions in photocatalytic process, as shown in the schematic diagram (figure 8*d*). The logarithmic plot over time of ZnO:Ag/rGO, ZnO/rGO and pristine ZnO along with corresponding linear fittings are shown in figure 8*b*. The correlation coefficient (*R*^2^) of ZnO:Ag/rGO, ZnO/rGO and pristine ZnO is determined and the values are 0.907, 0.914, 0.912, respectively, which are close to 1. It indicates that the decomposition process of MO with ZnO:Ag/rGO, ZnO/rGO and pristine ZnO follows first-order reaction kinetics (ln(*C*_0_/*C*) = *K*_a_*t* + *b*), where *K*_a_ defines the reaction rate constant. The photocatalytic activity can be estimated through kinetic rate constants of MO degradation. The obtained results (figure 8*b,c*, inset) show that the photocatalytic degradation rate of ZnO:Ag/rGO is found to be 0.01746 min^−1^, which is 2.7 times higher than reaction rate of pristine ZnO, suggesting that the recombination probability of excited charge carrier in ZnO is suppressed by the incorporation of Ag and rGO, which results in the improvement of photocatalytic efficiency. These results are in good agreement with transient photocurrent response of ZnO:Ag/rGO, ZnO/rGO and pristine ZnO.

## 4. Conclusion

The facile hydrothermal method was employed for the successful preparation of ZnO:Ag/rGO nanocomposites. The photo-electrochemical properties of ZnO:Ag/rGO were systematically investigated and results were compared with pristine ZnO. The Ag-doped ZnO nanorods embedded rGO composite is found to exhibit remarkable photo-electrochemical properties compared with that of pristine ZnO. The ZnO:Ag/rGO showed a high photocurrent generation of 206 nA cm^−2^ and excellent MO degradation (40.6%) under UV irradiation with the high reaction rate of 0.01746 min^−1^, because the addition of Ag and rGO improved the lifetime of photo-generated charge carrier by separating the electron–hole pair, which suppressed the carrier recombination in ZnO. The evidence for the enhanced carrier lifetime, carrier transportation and recombination was verified by time-correlated single photon counting, electrochemical impedance spectroscopic and OCVD measurements. The present study reveals that the ZnO:Ag/rGO nanocomposite can be used as an excellent photo anode for the electrochemical applications.

## Supplementary Material

Reduced Graphene Oxide Nano Composite for Photo-electrochemical Applications

## Data accessibility

The datasets which are supporting this article have been uploaded as the electronic supplementary material.
