# Supplementary material for: Ag-doped ZnO nanorods embedded reduced graphene oxide nanocomposite for photo-electrochemical applications
Source: R Soc Open Sci. 2019 Feb 6;6(2):181764. doi: 10.1098/rsos.181764 (PMC6408384; doi:10.1098/rsos.181764)
Supplement: Reduced Graphene Oxide Nano Composite for Photo-electrochemical Applications [file rsos181764supp1.docx]

**Supporting Information**

**Ag doped ZnO nano rods embedded reduced graphene oxide nano composite for photo-electrochemical applications**

Farheen Khurshid,^a^ M. Jeyavelan,^b^ M. Sterlin Leo Hudson^b^* and Samuthira Nagarajan^a^*

a. Department of Chemistry, Central University of Tamil Nadu, Thiruvarur, India.

b. Department of Physics, Central University of Tamil Nadu, Thiruvarur, India.

† Corresponding Author: msterlinleo@cutn.ac.in, +91-9486860214 (MSLH);

snagarajan@cutn.ac.in, +91-9443046272 (SN).

**1. Synthesis of Graphene Oxide (GO)**

The graphite was oxidized by using modified Hummer’s method. ^[1]^ In which 5g of graphite powder and 2.5g of NaNO_3_ mixed with 108 ml of H_2_SO_4_ and 12 ml of H_3_PO_4_. Thereafter, the mixture was stirred in an ice-cooled bath for 10 min. A 15 g of KMnO_4_ was gradually added into the above mixture under constant stirring and then the solution temperature was maintained at 5 ⁰C. The suspension was then allowed to react for two hours in ice bath and then one hour at 40 ⁰C. Afterwards, the temperature of the solution was raised and maintained at 98 ⁰C for 1 hour. To control the net reaction, 400 ml of deionized (DI) water was added into the suspension. After 5 min 15 ml of H_2_O_2_ was added into the suspension. Finally, the reaction product was filtered with G4 sintered crucible and washed repeatedly with deionized water and 5% HCl solution until the suspension becomes neutral (pH = 7). The final product was collected and dried at 60 ⁰C in a hot air oven.

**2. SEM Images**


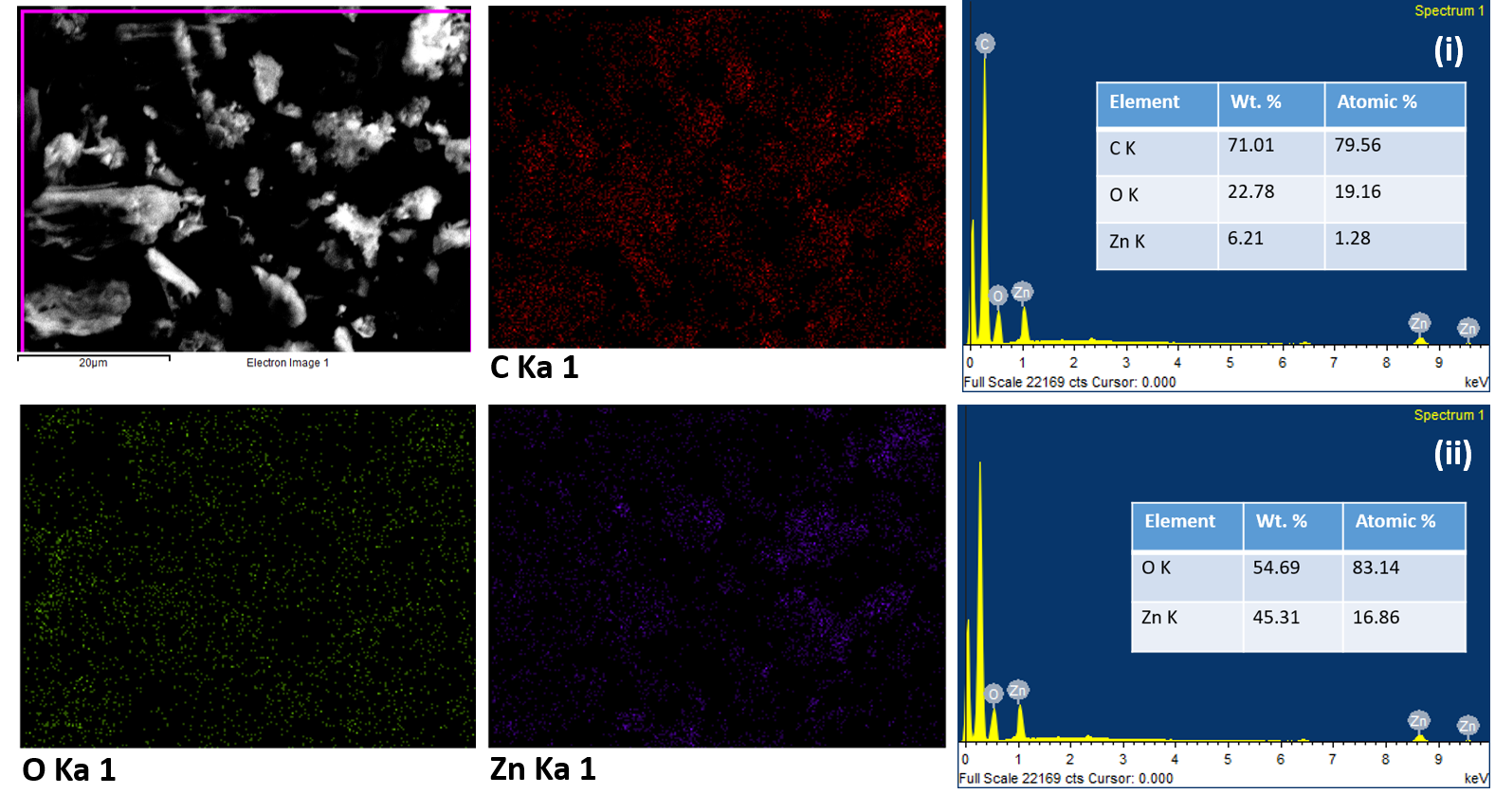


**Figure S1**. SEM morphology, elemental mapping and EDAX spectra of ZnO/rGO, atomic and weight percentage of the elements in ZnO/rGO were calculated with C and O (Figure S1(i)), without C and O (Figure S1(ii)).

**3. TEM Images**

**
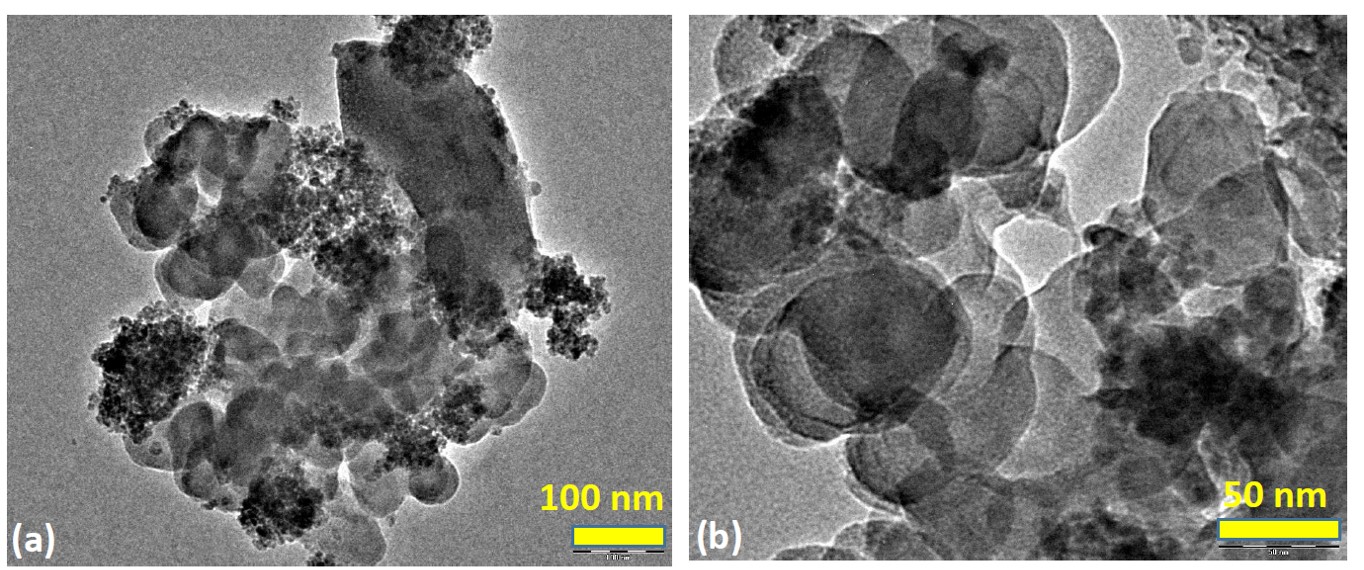
**

**Figure S2**. TEM images of ZnO/rGO nano composite.

**4. FT-IR Spectra**

**Figure S3**. FTIR Spectra of graphite, GO, rGO, ZnO and ZnO:Ag/rGO.


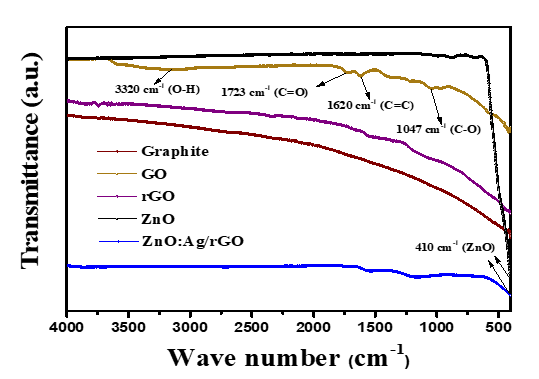


**Figure S3** shows the FT-IR spectra of a graphene oxide and the composite samples. The GO spectrum indicates the broad 3320 cm^-1^ corresponding to the hydroxy group (OH) on the surface of GO, 1723 cm^-1^ corresponding to the carbonyl group (C=O), 1620 cm^-1^ attributed to the unreacted domin peak and 1047 cm^-1^ attributed to the epoxy group of the GO. After the thermally reduced the GO majority of functional groups are vanished as a result of thermal treatment of GO. ^[1]^ The ZnO:Ag/rGO shows the weak absorption peak at 410 cm^-1^ which is attributed to the ZnO presence in the composite.

**5. Photocatalytic MO degradation**

Figure S4. UV Spectra of (a)ZnO (b)ZnO/rGO,and (c) ZnO:Ag/rGO

**Reference**

1. F. Khurshid, M. Jeyavelan, K. Takahashi, M. S. L. Hudson, S. Nagarajan., *RSC Advances* **2018,** **8** (36), 20440-20449.
